# An Investigation on the Nature, Growth, and Formation of the Human Teeth, with an Explanation of the Causes of Their Decay; Particularly of Its Uncommon Prevalence in the Middle and Northern States of America

**Published:** 1840

**Authors:** Horace H. Hayden

**Affiliations:** Baltimore.


THE AMERICAN JOURNAL

OF

### DENTAL SCIENCE.

Vol. I—No. VII, VIII.

[Aug. Sept. 1840.]

### AN INVESTIGATION

*On the Nature, Growth, and Formation of the Human Teeth, with an Explanation of the Causes of their Decay; particularly of its uncommon prevalence in the middle and northern states of America.*

(From the Medical Repository, for 1813.)

[ BY HORACE H. HAYDEN, ESQ. OF BALTIMORE. ]

Sir,—

During the short interview I had with you in this city, I mentioned to you that I had noticed a substance entering into the composition of human teeth, and those of animals, situated between the enamel and bone: and apparently differing in substance from either; that in the case in which I first discovered it, (which was a very large human tooth, that had gone in the most rapid manner to decay;) the appearance of this substance was similar to that of the membrane which lines the shell of an egg. I also requested of you, some information as to the most probable means by which to separate those substances, the enamel and bone, leaving the intermediate one entire.

Since which, I have tried various experiments with acids and alkalies without effect. Still, however, unwilling to give up the pursuit, I adopted a different plan, by which, in the course of the winter past, I have happily succeeded in my efforts, and by which I am enabled to present you with a beautiful display of the manner in which the enamel is united to the bone, and also of the substance itself which constitutes this union.

This I have been able to accomplish by the following method : having observed that this substance was in a degree elastic, I had reason to believe that it was more vascular than bone, and therefore susceptible of absorbing more fluid, consequently, capable of greater expansion ; and therefore, when exposed suddenly to violent heat, liable (like wet parchment) to sudden contraction, which must inevitably break up its attachment to the bone.

Under this impression, I subjected a quantity of pieces of the tooth of the Hippopotamus, and also the teeth of several animals to maceration in water, until they were completely charged through the whole substance. I then prepared a violent heat in a stove grate, and suddenly exposed the several pieces of various sizes, to its operation while still wet. The effect was such as gratified my most sanguine expectations. The violent heat conveyed from, or through the enamel to this intermediate substance, occasioned a sudden contraction, which broke up its adhesion to the bone, and in many instances to the enamel also, which affords a complete display of the intermediate substance entire, and separate from either enamel or bone, of which I have pieces of various sizes. This experiment must be well timed to insure a favourable result, on handsome specimens, particularly on human teeth, those of cows, &c.

Hence, I am happy in having it in my power, to submit to your examination, specimens of various forms and dimensions of all three, viz. the enamel with the intermediate substance attached to it, the intermediate substance alone entire, and also attached to the bone, which I flatter myself will be sufficient to put the matter beyond the possibility of doubt, as to the correctness of my opinion on this subject. It may be asked, of what service is this fact apparently so unimportant ? Or what purpose is answered by it ? I shall not, at present, attempt to detail at large, the advantages which I am inclined to think it affords ; but this much I will venture to advance, viz. by it, we advance one step further in the true knowledge of the mysterious operations of some of nature's works ; and thereby are enabled to embellish with genuine truth the interesting pages of *Natural History.*

From the diversity of opinions, advanced by different authors, on the structure and formation of teeth, some of which are ingenious, others much less so, yet supporting an equal claim to correctness and truth, I have been induced to offer some remarks on this head.

This I the more readily perform, since they are the result of my own observations, and actual experiments. Therefore feeling a consciousness of their correctness, I shall cheerfully submit them to your perusal, and candid decision.

To Dr. Mitchill, &c.

Until within the last century, little notice seems to have been taken of the subject, as it relates more particularly to the teeth of the human species, but since the commencement of which, numbers have been engaged in an inquiry into the subject with a degree of zeal and success that has nearly kept pace with the improvements in the other branches of relative science.

Among those, various have been the opinions advanced on the subject generally ; but as it respects the formation of the human teeth, and those of some of the inferior order of creation, I know of no one that has carried his researches further than Dr. Blake of Edinburgh.

But with what degree of accuracy he has applied his remarks in the course of his inquires I shall not, in the present instance, take upon myself to declare. But since some of the opinions he has advanced in his publication of 1798, and 1801, (printed at Dublin,) are irreconcileable to my ideas, particularly those which relate to the formation of the enamel of the teeth, and that he terms the Crusta Petrosa, I shall take the liberty of offering some remarks, not however with a view of refuting his opinions; but to state what appears, at least to me, to be more consistent with probability; and thereby, if possible, rendering the subject less questionable.

In treating of the human teeth, and particularly of the sacs, or membranes that surround the pulps, he observes, "the internal part of these sacs, soon after the commencement of the bony shells, deposits on their points and sides a soft earthy matter, moistened with a mucilaginous fluid; a small quantity of which is found between the membranes and shells." Further, " It may be proper to remark that it is *this soft earthy matter* which afterwards becomes the *cortex striatus*," or enamel.

That this beautiful provision of the membranes, is made to accompany the pulps of the teeth, no doubt can be entertained; but, that nature should have recourse to this mode of economy in order to perfect her works, seems to me to be inconsistent with her general plan.

That the periosteum, or membrane, constitutes the medium through which bone is deposited and formed, is an opinion long since advanced, and I believe, long since refuted.

That all the membranes in the human system are formed by the same means, and from the same source is very certain; that they receive branches from the same arteries, veins, and nerves as the other parts of the system, and are supported by the same living principle, is equally as conclusive. Hence, if bone is formed by a membrane in one instance, we may reasonably infer that it is in every instance deposited and formed by the membranes or periosteum.

We know that the chick within the egg, is likewise enveloped in a beautiful membrane, immediately within the shell; but we have little reason to suppose that the membrane has any agency in depositing the bone cellular substance, and much less, the feathers which differ as much in consistence from the general mass of the chick, as the enamel does from the bone of a tooth.

In conformity with this principle, we might expect, that the external table of the cranium is deposited by the pericranium; and, since the principle of action is simultaneous throughout the membrane, that ossification, instead of commencing at several points, would manifest itself at the same time, and at every point throughout the whole internal surface of the membrane, and progress with equal uniformity, until the external table is in a degree perfected, after which, the dura mater ought to take upon itself the same office to perfect the internal table; but this, I believe, is not the case.

There are numerous instances in the wide range of nature which can be adduced, that will operate with equal, if not greater force, against the doctrine, that bone is deposited from, or by, either the periosteum, or membranes; since which, much less am I inclined to believe that the enamel, or, cortex striatus, is deposited by any particular membrane under any degree of action, or modification, to which they are naturally liable.

We are not to conclude, because the enamel differs in texture and consistence from the bone of a tooth. that a membrane, subject to any peculiar change of action is necessary, to deposit and perfect the enamel ; or in fact any other substance, which may be naturally appended to men or animals differing, in like manner, in its character.

Can we suppose that a membrane is necessary to deposit, and perfect the lofty anthers of the stag ? or the horn-like teeth, which sometimes twenty or more in number enter into, or rather grow from that appendage which characterizes the saw fish ? or, those curious appendages attached to the tail of a rattle snake ? or, the whale-bone-like tuft, growing from the breast of a male turkey ? Can it be even supposed that the beautiful convolutions of the turbinated bones of the cow, horse, &c. which resembles a spiral roll of rich Brussel's lace, is deposited by a membrane ? I presume not.

But this mode of reasoning only serves to shew, that I do not rightly understand, or comprehend the subject; therefore, I will proceed to notice some futher observations contained in his ingenious essay.

In the formation of the teeth of different animals, Dr. Blake seems to have discovered the same prevailing economy to exist, as it respects the formation of, or disposition of the enamel, or cortex striatus, by a membrane; and also some other modifications, and other operations that prevail during the growth of teeth, not hitherto so particularly noticed, and illustrated; but, which at the same time, seem to be by no means free from exceptions.

In describing the formation of the grinders of a fœtus-calf of three months old, he observes, "the membranes which are intimately connected with, or *derived from* the *internal* part of the *gum*, can be easily separated into two lamellæ, the external of which is very vascular, though I could not trace a single blood vessel entering into the internal one. These membranes surround the pulp ; and duplicatures of them pass down between the internal divisions of it, exactly similar to the passing of the pia mater between the convolutions of the brain.

"As soon as the bony shells are perceptible, the internal part of the membrane secretes and deposits on their points, and progressively on their sides a soft earthy matter, similar to moistened chalk, and is itself moistened with a mucilaginous fluid, a great quantity of which is found between the membrane and shell." This soft earthy matter gradually assumes the crystalline appearance of the cortex striatus.

With regard to the membranes being connected with, or derived from the internal part of the gum ; and which, is also the case with the membranes of the human teeth, agreeably to Dr. Blake's observations, it seems needless to offer any comments ; since he is so candid as to make the following acknowledgment :—

" Having repeatedly inspected and examined the jaws of young subjects, I observed blood vessels passing from the gum to the membranes, destined to form the *cortex striatus.* It was natural enough then to conclude, that as the investing membranes were derived from the gums, their nourishment too did originate from the same scurce, and that the use of the vessels which entered the proper foramina of the jaws, was only to form the pulp and bony part of the tooth.

" Futher considerations of the subject, however, urged me to *relinquish this opinion ;* it would by *no means hold good* on comparing it with what takes place in the jaws of large animals."

But with respect to the duplicatures of the membranes which surround the pulp, being sent down to form the cortex striatus around those conical processes which are discoverable in the grinders of the cow or ox, the sheep, deer, camel, &c. I must confess myself, not unwilling, but unable to reconcile it to my present opinions and views, of the formation of the teeth of man, and animals.

Dr. Blake has certainly treated this part of the subject, as it relates to both, with a degree of ingenuity that does not fall to the lot of every one. But, however far I may have fallen short of it, I must beg leave to examine the last mentioned circumstance, on the principles he has alleged and supported.

We are led to believe, that the first point, or points of ossification upon all teeth, as far as we are acquainted, is on the tops of the pulps ; and that it progresses towards the termination and completion of the roots or fangs, and that, as ossification progresses, a substance is discoverable on the surface of the body, or crowns of the tooth, which, as Dr. Blake correctly observes, resembles moistened chalk, and which in this state may be easily scraped off with the finger nail. This soft earthy matter gradually assumes the crystalline appearance of the cortex striatus, and without doubt, is what forms the ground work or basis of the enamel.

But the question is, whether this chalky substance, or the real cortex striatus, is deposited and formed by the internal membrane.

Dr. Blake observes, page 81, that, " as soon as the *bony shells* are *perceptible,* the internal part of the membrane secretes and deposits on their points, and progressively on their sides, a soft earthy matter similar to moistened chalk, &c. The cortex striatus is *first perfected* or crystallized on the *cutting edges* or *protuberances* of the tooth, and proceeds gradually from thence to the neck, when it terminates ; and in *proportion* as the first part of the cortex striatus is crystallized, *that portion* of the *membrane* which formed it, becomes thinner, less *vascular ;* and at length, having performed the particular function for which it was destined, is totally wasted or absorbed ; *part* of the membrane still remains on the body of the tooth, this however, is wasted as the cortex striatus covered by it attains to perfection."

In the first instance, as it relates to the formation of the enamel, or cortex striatus of the human teeth, I would ask if it is probable, or even possible, that this internal membrane could perform its functions duly in depositing the enamel of a tooth, after that part which forms the apex of this little capsule is wasted and obliterated ? What is its situation ? It is within the external membrane not adhering to, but loosely surrounding the body of the tooth, and within which is contained a mucilaginous fluid. And what are its connexions ? It adheres firmly to the neck of the tooth, and is slightly connected with the external membrane, but contains no blood-vessels capable of carrying red blood ; at least I could not discover any, although assisted by a very subtle injection.*

This being the case, is not the medium of circulation in the membrane in a degree deranged by a part of it being wasted ? If so, by what is the remaining part of the membrane nourished so as to perform its office in perfecting the enamel, since it adheres only to the neck of the tooth, and slightly to the internal membrane ? It cannot derive it from the bone of the tooth, to which it adheres, or is attached ; and it appears improbable that it derives it from the external membrane since it is destined, as will appear hereafter, to deposit and form a very different substance agreeable to Dr. Blake's opinion.

In the next place, if the same order is observed, in the formation of the teeth of various animals, viz. the cow, sheep, deer, camel, &c. as to the commencement of ossification at the tops of the pulps, and that as the bony shells are perceptible, the internal part of the membrane secretes and deposits, what afterwards becomes the cortex striatus, and that, as it is formed, the membrane is gradually wasted, how, or in what manner is the enamel, or cortex striatus perfected round those conical processes which are seen in the grinders of those animals, and which sometimes extend down almost to the bifurcation of their roots ?†

If ossification commences at the top of the pulp, and if, as it progresses, the enamel is deposited, and as it is perfected the membrane is wasted, is not the continuity of the duplicatures interrupted and cut off ? It may be said not ; for the duplicatures of the external membrane descend with it, forming a kind of hollow inverted cone, and that this is sufficient to support it, until the office of the internal one is perfected. In

---

* At page 81, Dr. B. observes, "I could not trace a single blood-vessel entering into the internal one."

† Those processes in the superior grinders of the horse, extend down sometimes three inches : but others quite to the bifurcation of the roots.

fact, we are left to believe, or at least to suppose, that the offices of the pulp, and surrounding membranes are performed in regular succession to each other, and that the action of the membrane is suspended until the tooth is nearly completed; for Dr. Blake observes (page 81) that "the duplicatures extend downwards, until checked by the junction of the internal *plates* of the *shells* which thus separate them from the pulps."

From the use of the terms "plates of the shells," we may conclude, that by it, is meant bony plates. In this case, as those conical processes extend down nearly two thirds of the body of the grinders of the cow, horse, &c. we may infer that ossification has progressed thus far before the internal duplicature begins to deposit the enamel.

But this appears to me to be inconsistent not only with the general economy of the teeth, but with his own principle or theory.

But, in reverting to the subject, we have reason to believe that the continuity of the internal duplication is intercepted, and its action destroyed, from the following circumstance. Dr. Blake observes (page 82) "that the protuberances of the tooth first appear, and thus *intercept part* of the *nourishment* from the duplicatures of the membrane which passed down into the internal cavities."

When the tooth rises somewhat higher *all nourishment* is cut off from the internal duplicature of the membrane which is then found *dead* and of a *darkish purple colour*. Can it be admitted, if the enamel is deposited by the membrane, that it can be duly perfected when the membrane is at first partially destroyed and gradually progressing towards a total wasting away?

We are further led to a conclusion that the circulation and action in both the internal and external membranes are cut off from the following:—

Dr. Blake observes, (page 82) that "it appears that the internal part of the membrane secretes the earthy matter of the cortex striatus, and that as soon as it has performed its function it is wasted or destroyed; for its external lamella, as soon as the *upper part* of the cortex striatus is crystallized, begins to deposit on its surface a substance differing from *either* the *bony part* or the cortex striatus, being harder and more brittle than the former, and less so than the latter;" this substance, for "distinction's sake," Dr. Blake calls the "crusta petrosa."

There is every reason to believe, that a certain degree of uniformity prevails in the action of the vessels that deposit the bone of the tooth, the enamel, or cortex striatus, and the crusta petrosa; and that when ossification is begun on the pulps, the points or shells unite, and the other substances are in due order deposited, until the top of the crown of the tooth is completed in its form.

Hence it must be admitted, that the upper part or orifice of those conical processes, when the duplicatures are sent off, are in most instances filled up by the crusta petrosa, before the tooth is formed down to the termination of those processes, or at least, to the bifurcation of the roots; consequently, the duplicatures, or membranes, must be intercepted.

Of this, we have what I consider the most unequivocal proof, in examining the grinders of the animals before mentioned. It appears obvious, that nature often varies in her operations, so much so, as to lead us to form conclusions directly opposed to each other in examining different subjects of the same class and order.*

Hence there remains no doubt, but that the subject of Dr. Blake's observations, and from which he drew his conclusions, were such as to authorize him in a degree to advance the following opinion on this head.

"In some few instances, the grinders remain sufficiently long under the gum, so as to allow the cavities in which the membranes passed down, to form the cortex striatus to be *nearly* filled with the crusta petrosa. In most cases, however, these cavities are filled entirely with the matter of the food, by the broken particles of the teeth, whilst grinding, or by sand, clay, &c."

In examining the grinders of the animals before mentioned, of different ages, we see numerous instances, such as described by Dr. Blake; but their number, as far as my observation has extended, falls far short of those which are, on the contrary, completely closed up with bone or crusta petrosa.

Hence, I am induced to believe that the duplicatures of those membranes passing down into the bodies of these teeth, to form the conical processes, are in most instances interrupted, and the nourishment cut off by the process of ossification being completed at the top of the tooth first; thereby leaving the lower, and greatest part of these processes an entire void, or destitute of the crusta petrosa; for, in making a longitudinal section, that shall intersect the conical processes of the grinders of either the cow, horse, &c. we find, in three cases out of five, if the animal is young, the upper part of the process filled with bone or crusta petrosa as it descends, it has less the appearance of bone; thus it gradually assumes the appearance of pure phosphate of lime, wanting the gelatine to give it consistence; from thence it assumes a brownish, then a purple colour, and terminates in many instances, in a dark or blackish substance resembling dried blood, which no doubt, (particularly in the grinders of the cow, or ox) appears to have more the consistence of bone, possessing more of the gelatine or gluten than lime, all of which I shall endeavour to account for hereafter.

That these conical processes are completely filled up at the top of the teeth, and consequently the nourishment of the duplicatures intercepted, leaving the remainder of the processes hollow below, will appear evident from the following.

---

* Cuvier observes, in his treatise on the horned rhinoceros, that "to obtain a complete knowledge of the teeth of herbivorous animals, it is not enough, to have seen them at one period of life, as these teeth are continually wearing down; the figure of their crown is also in a continual state of change; and the naturalist must follow them from the moment when they pierce the gum, to that when they fall out of the mouth."

In describing the process of ossification in the grinders of the elephant, and pointing out the different appearances which are manifested in the duplicatures that are sent off to form the cortex striatus and crusta petrosa, Dr. Blake observes : " This beautiful contrivance was necessary, otherwise, as the processes of the pulpa elongata, and the bony shells extend downwards on them *many inches previous* to the *junction* of their edges, the upper part of the cavity or *interstice between them* would be filled by the crusta petrosa, whilst the lower part of it would be empty, *which sometimes,* though seldom, happens in the upper grinders of the horse."

On examining the grinders of those animals, at least the cow and horse, at the period when their grinders are formed and nearly all appear through the gums, I have found in most instances as far as my observations have extended, that the processes were filled up, and the teeth completely formed. In other instances where the animal was more advanced in age, and where the grinders were abraded, or worn down by mastication, those hollow processes were just making their appearance. And again in other instances, when still more advanced in age, the grinders are so far worn down as to expose the remainder of those processes completely hollow, and (in which Dr. Blake is perfectly correct) filled with extraneous matter.

Of such, I am inclined to believe he drew his conclusions ; and in fact there are instances even in young animals, which if examined separately would strictly justify his opinion on this point ; for I have seen in examining the grinders of the horse, the fifth molaris from the anterior one in the superior jaw with its conical processes entirely open at the grinding surface, but nearly filled with extraneous matter ; in like manner was the corresponding one on the opposite side of the jaw, while the four anterior grinders, and the sixth or posterior one were entirely filled up with bone; and (being a little worn down) presenting surfaces with all the beautiful convolutions of the external plates of the cortex striatus, and also the internal plates surrounding the conical processes, which might have, when considered in relation to the grinders of the opposite jaw, suggested, certainly no inappropriate idea of the manner in which the grinding surfaces of a pair of mill-stones ought to be pecked in order to perform their office duly.

Cases of the last mentioned description I consider as coming under that class which Dr. Blake has described, in which ossification has progressed more rapidly, perhaps, than at any preceding, or subsequent period, during the dentition of the animal ; in consequence of which, the tooth, or teeth, were forced out through the gum, and the nourishment cut off from the duplicatures that deposit the bone, or crusta petrosa in the internal processes before it had sufficient time to deposit it, except at the top of the tooth which was quickly worn down, leaving the cavity to be filled with extraneous matter as before.

Instances of this kind, in which ossification seems to progress more rapidly than at other times is often manifested in the economy of the teeth. Not only so, but we see it in other instances of the animal economy; but in none, however, more evidently than in infants, where, in some cases, the bregma is closed up in eighteen months, or less; in others, it remains open for three years, or more; a circumstance which in my opinion affords room for some very important and useful deductions relative to the action of the vessels in depositing ossific matter under particular circumstances. But this is wandering from the subject.

Having cursorily noticed the opinions advanced by Dr. Blake relative to the formation of the enamel, or cortex striatus, and crusta petrosa, by membranes which surround the pulps of the teeth of man, and animals, duplicatures of which are sent down to form the conical processes in the teeth of the latter: I must beg leave, in the next place, to examine in a similar manner the grinders of the elephant.

Not having had the pleasure of seeing a dissection of one of those monstrous animals, nor of an opportunity of injecting the pulps of any of their formidable teeth; in whatever instance I may fail for want of ocular demonstration, I must pattern from *example* and resort to conjecture to supply the deficiency. In describing the strucure and formation of the grinders of the elephant, Dr. Blake *seems* to have discovered the same mode of economy prevailing as to the formation of the cortex striatus and crusta petrosa as that which is manifested in most other graminivorous animals, with the exception of some small deviation, for he observes:—

"I mentioned to Mr. Corse, in Edinburgh, that the pulp of an elephant's grinder was divided into a number of conical processes, and that duplicatures of the investing membranes passed down between these processes to form the cortex striatus. That ossification *commenced* on the *highest point* or *points* of these processes, and formed conical bony shells on them which extended *downwards* for *many inches previous* to the *junction of their sides.*"

From the exact representation of a section of the elephant's grinder which Dr. Blake has given us, and from an examination of a very large and beautiful grinder which I have in my own possession, there is every reason to believe that he is so far mostly correct. But how, or why he should so soon throw himself off his guard, and immediately advance an opinion, which if not contradictory, is so fully calculated to render the above opinion doubtful, is to me a circumstance not a little surprising. For he observes "that as soon as the bony shells were perceptible, the internal lamella of the investing membrane began to deposit the soft earthy matter of the cortex striatus on their external and *upper* surface, and that *as soon* as it was *crystallized* on the *upper part* of the *shells*, the investing membrane assumed a *different mode of action* and *began* to *deposit* the crusta petrosa on *their points* and *sides*, in order to fill up their interstices or spaces between the shells where the membranes passed down to form the cortex striatus, in a similar manner to those of the horse.

Now if those conical bony shells extend down several inches *previous* to the *junction of their sides*, which is a fact no one can doubt that has seen them; if, as soon as ossification had commenced, and the bony shells or tops of those processes were perceptible, the earthy matter of the cortex striatus was deposited on them; and that as soon as it was crystallized, the external or investing membrane begins to deposit the crusta petrosa; is it possible, or can we even suppose, that these conical processes, which are in some cases six inches in length, would have remained separate from each other in their whole length? If this investing membrane assumes its action, and deposits the crusta pretrosa so soon after the commencement of ossification, and also the crystallization of the enamel, or cortex striatus, would it not probably fill the space of one quarter or three-eighths of an inch in thickness (which is the greatest distance I have ever seen between those processes, at least at the grinding surface) with bony matter, or crusta pretrosa, as soon as four or five inches of bone is formed in length downwards, consequently unite those processes at their tops, probably before they were formed half their length downwards? Yet we see it is not the case, but on the contrary they are formed, and remain separate from the top almost, or quite down to where the sides of the shells and cortex striatus unite, and where the formation of the roots of the grinder commence.

In support of this, we have not only the representation of a section of a growing elephant's grinder which Mr. Corse politely presented to Dr. Blake, of which the two posterior processes are at least represented entirely separate from each other, and from the main body of the grinder: but we have also the opinion of Dr. Patrick Blair,* (as quoted by Dr. Blake) who observes, "several of which rudiments he observed lying stratum superstratum, or placed perpendicularly across the saw; they were two or three times intersected by membranes, whereby they could be disjoined; but after he had taken out several of those rudiments or processes, he observed no more such separations. Where the ligaments or membranes cease, they become extremely solid and ponderous."

Having cursorily noticed *some* of the exceptionable parts of Dr. Blake's theory, I shall in the next place, and in as brief a manner as is consistent with the nature of the subject, proceed to explain to you my own ideas on this head; taking the liberty at the same time, of pointing out to you some important relations which present themselves as analogous and highly interesting.

I have demonstrated to you, as well as a number of respectable scientific characters, the existence of a substance entering the composition of teeth, differing apparently in consistence from the other substances, (viz.) enamel and bone, which constitutes the medium of union between those two substances. I feel myself justified in rejecting most of the opinions hitherto advanced, on the formation of the enamel of teeth, and particularly that which supposes it to be deposited and formed by a membrane.

Mr. Home (in treating of the pulps of teeth, page 4,) says: "This pulp is enclosed by a capsule, the cavity of which, while the tooth is growing, is filled with a viscid fluid similar to the synovia of joints, and

---

* Who dissected an elephant that died at Dundee, in Scotland.

this fluid, by the absorption of the thinner parts becomes inspissated to a proper state for crystallization, so as to form the enamel which adheres to the surface of the tooth. Dr. Blake observes, 'I shall just ask Mr. Home what deposits other mucilaginous fluid?—I answer for him, the investing membrane.'"

With due submission I beg leave to ask either or both of these gentlemen, what it is that deposits the *semi osseous strata* between the enamel and bone of the tooth?

I shall not be so positive in saying, at least that it is deposited by *any* membrane, since there must necessarily be a third, and no such has been discovered.

Therefore, the whole must rest upon mere conjecture. But were I to cherish a belief, that nature has formed a membrane in the whole animal economy destitute of blood vessels, and capable of depositing and forming the enamel, cortex striatus, I should remain in ceaseless anxiety lest the crystalline lens of my eyes should become a mass, similar in substance to the enamel of a tooth; or, that some of the membranes of the eye (which are doubtless subject to the same laws of action as other membranes) should deposit a like substance, which would ever after deprive me of the indescribable pleasure of contemplating the beauty of her inimitable works.

Dr. Hunter seems to be of the opinion that the enamel of a tooth, is the earth more fully depurated or strained off from the common juices.

This conjecture seems to be by far the most plausible, and one that is strongly supported by the following consideration, whether he had it in view or not.

It appears from a strict analysis of both the bone, and enamel of the teeth, that they contain both phosphate and carbonate of lime; though in different proportions.

Mr. Hatchetz says: "The enamel appears, only to be different from tooth or bone, by being destitue of cartilages, and by being principally formed of phosphate of lime cemented by gluten."

Now as there is no instance, as yet ascertained, in which those substances are known to be deposited by a membrane, destitute of blood-vessels, and that on the contrary they are both deposited by the blood-vessels (as will be made to appear hereafter) it follows, since the internal membrane surrounding the pulp contained no vessels capable of carrying red blood, that the opinion of Dr. Blake remains very doubtful, while that of Dr. Hunter appears, not only possible, but very probable. That the enamel is the real production of the tooth, or rather, that it is secreted or deposited by it, appears probable from the following circumstance.

The texture of the osseous part of the tooth of the hippopotamus is much finer than the tusk of an elephant,* yet much coarser than that of the tooth of the common horse, and still more so, than that of the cow or

* I do not mean to infer that the tusk of an elephant has enamel upon it.

ox, buffaloe, &c.—The enamel differs in like manner.—That upon the tusk of the hippopotamus is perhaps the coarsest in its texture of any, at least that I know of; that on the tusk of the horse approaches nearest to it, except that on the grinder of the elephant.—The osseous part of the teeth of the buffaloe, cow and dog, resembles, in its texture, that of the human teeth, in the strictest sense, and so likewise is the enamel.—There is not a human tooth in nature which presents a finer and more beautiful enamel than is found on the cutting teeth of either of the last mentioned animals; hence, the beauty and texture of the enamel seems to depend on the texture of the bone which it covers. Whereas, if the synovial fluid of Dr. Home, and which, according to Dr. Blake, serves to moisten the chalky substance, which afterwards becomes the enamel, is secreted from the membrane, we have much reason to believe, that this substance upon all teeth would be alike in beauty and fineness; on the contrary, it differs very materially.

It may be said, that the larger the animal, the grosser the membranes and vessels, and therefore the coarser the enamel; but the sheep and the hog are much smaller animals than the cow; yet the enamel on the teeth of the former is much inferior to that on the teeth of the latter. As to the bone of the tooth serving as a foil, it is out of the question; for the bony part of the tusk of the hippopotamus is whiter than either, while the enamel is still the coarsest. In the next place, if the enamel was deposited by the internal membrane, upon the bony substance of the tooth, it would, after being perfected, be liable at all times to scale off through a deficiency of the uniting medium.

This will appear evident when we consider the formation of the crusta pretrosa.

This bony substance of the tooth is first formed at the top. As ossification progresses, the enamel is formed, and as it is perfected, the crusta petrosa is deposited; but *no union* of those two last substances ever takes place, except such as is formed by the inequalities of the crusta petrosa impressing itself in the most minute manner when soft, into the inequalities of the surface of the enamel. Now if the enamel was, or is, deposited by the membrane upon the bone of the tooth, and by a membrane also, the crusta petrosa is deposited upon the enamel, we have every reason to believe that the union of the crusta to the enamel, would be as intimate as that of the enamel to the tooth.

On the contrary, as I before observed, it is never the case; the crusta petrosa will in all cases flake off from the enamel, leaving it entire and beautifully white—whereas, the enamel is so intimately united to the tooth that it cannot be separated by any violence (except by the method I have adopted) without splitting up the bone also.

Lastly, it appears that in the formation of the teeth, this chalky substance is deposited upon, and by the teeth; that it is this substance which, when formed constitutes the *semi-osseous strata* which I have demonstrated to you, and which is to be seen upon every tooth, where there is an enamel, and that it is through this semi-osseous strata, or by it that the more subtile parts of the ossific matter of the tooth is infiltrated, strained off, or deposited, which, when crystallized, forms the enamel or cortex striatus.

This opinion is rendered the more probable, and is fully illustrated by the following considerations.

Whoever will consider the difference between the texture of the enamel, and bone of a tooth, will readily grant, that it is next to impossible, that these two substances could ever be so intimately united, without the agency of this, or some other intermediate substance.

Secondly, that in the living tooth this *semi-osseous strata* is organized and possesses a living principle, and is necessarily so, or it would by violence cleave from the tooth, (since it is organized) bringing the enamel with it.

Thirdly, it is necessarily endowed with the living principle in order to support and nourish the enamel, which would otherwise become dead, discoloured, and fall away by action of heat, and acrid secretions, which at *all* times prevail in a greater or less degree in the mouth.

This, I am sensible is directly opposite to the opinion of Dr. Blake, who maintains, that after the enamel is once formed or perfected, it receives not the slightest degree of nourishment.

On this particular point, a few observations, I hope, will not be considered unseasonable.

That the enamel, which is supported by the semi-osseous strata, is nourished, is not only a natural conclusion, but demonstrated from the following circumstance. Whenever any acrid matter penetrates the enamel, at however small a point, and near the gum, it soon spreads, destroys the living principle in the *semi-osseous strata*, its union with the bone of the tooth is broken up, and with it the enamel falls ; constituting no doubt, what is generally termed denudation; leaving the bone of the tooth underneath exposed to view.

It may be asked, since the *semi-osseous strata* is once exposed, why does not the destruction go on till the tooth is deprived of its enamel? With the same propriety, may be asked the following question; why does not gangrene, when first it makes its appearance, always go on till it destroys the patient :—I presume the following answer will suffice for both, (viz.) That by some modification or effort of the living principle, it overpowers, or subdues the diseased action, thereby setting bounds to its progress.

That the teeth not only possess a living principle, and are nourished, and the *enamel* also, must appear evident from the following :—

Dr. Blake acknowledges what is a well known fact, that, "If by chance the vessels which enter the roots, and are distributed on the pulp, be torn or destroyed, the body of the tooth becomes discoloured, sometimes nearly black."

Not only so, but human teeth artificially substituted in the mouth of a person, are subject to the same change, and in a few years become soft, but never cartilagious, and entirely unfit for use.

In this case the discoloured bone does not act altogether as a foil for the enamel, but the enamel itself is changed in colour; and as its point of union with the bony part is destroyed, it changes into dark brown spots, which at length fall off from the bone.

We have more complete illustrations of this fact in artificial teeth, made of the tooth of the Hippopotamus. This, like all other substances partaking of the quality of ivory or bone, is in a few years destroyed, when made into artificial teeth, and set in the human mouth. And likewise, in this case, not only the osseous part is gradually destroyed, but the enamel, or cortex striatus seems to keep pace with it, and changes into dark brown, or rusty spots, which in some instances pervade the whole enamel, and what is a little singular, if the bony part of the tooth becomes soft and almost black by long use, the bone may, by a particular process, be rendered almost as white as ever; but no process whatever, will restore the enamel to its primeval beauty when once those spots make their appearance. Besides, the part of the artificial tooth which is hollowed out to be fitted to the gums, leaves the superior edge of the tooth thin, and which is generally soonest pervaded by the *destroying agent.*

As soon as it penetrates the *semi-osseous strata*, or point of union of the enamel with the bone, the adhesion is destroyed and the enamel flakes or crumbles off, leaving the surface of the bone underneath smooth but discoloured.

Was not the enamel on healthy teeth in the human mouth to participate in the living principle inherent in the teeth, and consequently be nourished, it would ultimately fall into a similar state, and perish, which is never the case except partially, while the tooth remains duly organized and healthy.

Having offered my opinion on the formation of the enamel of teeth, with some occasional remarks, I shall proceed to explain to you my opinion also, on the formation of the crusta petrosa, which is said to be deposited by the external lamella, or membrane which surrounds the pulps of the teeth.

There is not perhaps, in the whole animal economy, so striking an example, so complete a specimen of the manner in which bone is deposited and formed, as is presented in the examination of the teeth of man and animals; which, had Duhamel or Smellie, (I forget which) attended to, he would have but little occasion to resort to the periosteum (in a natural state) to account for the manner in which bone is formed.

This I think will appear evident to you, on a view of the different cases which I shall adduce in the course of my remarks.

The crusta petrosa, which is to be found in the teeth of almost all graminivorous animals is said to differ in substance from bone, or the enamel of a tooth, being whiter and more brittle. On the body or crown of a tooth it is without doubt more brittle ; and for a very good reason. Where it is deposited upon the enamel of a tooth, it is cut off from all nourishment, therefore does not possess that tenacity which it otherwise would do. But on the roots of the grinders of those animals where it continues to be deposited for a time unknown, after the tooth is formed, it retains every characterestic, and is in reality, as perfect a one as there is in any part of the body ; and will, I presume, on a strict analysis, afford the same results, or nearly so, as that which is universally admitted to be real bone.

Hence, there seems to have been but little necessity of resorting to the membrane as the medium by which this substance is deposited, since it (the membrane) contains blood vessels, ramifications of which are distributed all over it.

Thus it is with the membranes which line the processes or sockets of the superior grinders of the cow, buffaloe, &c. Numerous little foramina are seen, through which pass small branches of blood vessels to be ramified all over the membranes lining the sockets. These little foramina (I do not mean canullæ) are very perceptible in the plate which separates the sinuses and antrum of the superior maxillary bone, from the teeth of those animals. (Here also I do not include the foramina through which vessels and nerves are sent down into the teeth to nourish them.)

Hence the constant secretion of bony matter, or crusta petrosa on the roots of those grinders, after the pulps had completed their offices in forming the teeth.

As the animal advances in age, we see little spiculæ shooting out from the sides of the roots, and corresponding exactly with these foramina, as if (the circulation becoming less vigorous, and there being but little probability of much longer usefulness) to close them up, as the period of the dissolution of the animal is approaching.

This of itself might seem sufficient to demonstrate the manner in which bone is deposited and formed by the action of the arteries ; but for a more complete illustration, I will return to the external lamella or membrane, duplicatures of which are sent down to deposit the crusta petrosa, in the conical processes as beforementioned. Here, instead, of bone (or crusta pretrosa) being deposited in these processes by the duplicature of the membrane, we have what I consider proof positive of its being deposited only by the arteries that are distributed upon the membrane. In describing the substance which is found in the conical processes of the grinders of different animals, Dr. Blake observes, " This adventitious matter remains quite *spongy*, and of a different colour and texture from any of the *other three* above described parts : In the cow, it is of a blackish, or dark brown colour. This spongy appearance is only manifest in those cases where the tooth is much worn down ; then it is, that we find this bone, or crusta petrosa less perfectly formed, and which becomes still less so in general towards the bottom, or termination of the processes, until, in some instances, as I before observed, it ends in a hollow cavity ; in others something resembling *dried blood*.

If we examine those processes critically, instead of finding them spongy, we see from four, to six, or seven very small holes, or foramina, arranged mostly in a line, at unequal distances, and following the centre of the curve of the process.

This appearance I shall account for in the following manner:—The bony substance is deposited by the arteries upon the internal sides of these hollow processes, or otherwise upon the enamel that surrounds them; as the space is filled up, and the sides approximate each other, twe two folds of the duplicature likewise approximate, until they are brought into perfect contact; by this time the space is filled with bone; but in consequence of ossification commencing at the top of the tooth (and also in those processes) and being then perfected first, the arteries and vessels are gradually interrupted. As the circulation is interrupted the blood is thrown into the vessels below, without the power of returning. As the tooth is completed above the arteries and vessels, are entirely cut off, and no appearance of them to be seen; but as the tooth wears down, those little foramina appear in the order we see them, and which were doubtless once occupied by blood vessels; for in examining the section of a grinder, we can trace those holes through the body of it, still filled with a substance, in appearance like dry blood—while the bony substance in the lower part of those processes is likewise tinged sometimes with a reddish, brown, and almost black colour, as Dr. Blake observes.

We sometimes see in the superior grinders of the horse after being somewhat worn down, two of those little foramina, one and sometimes two in the centre of the conical process, which can be traced quite to the bottom of it, having the same appearance as those above described.

It may be said, that these are unimportant details, and merely accidental, having no relation to the subject of ossification. But I cannot help viewing them in a very different light, and whatever they may appear to be in the opinion of some, they appear to me as letters or characters in nature's great alphabet, which, when combined form syllables and words by which we can read the description of some of her secret operations.

As another instance in which bone is deposited by the arteries, I shall adduce the following. It is said by almost every author who has written on the diseases of the teeth, that there is one to which they are liable, which is termed a swelling of the fangs or roots.

That there is an inflammation and thickening or swelling of the investing membrane of the fangs, I will admit, nothing is more common; but that there is a swelling of the fangs, I am not so readily inclined to believe.

That there is sometimes an enlargment of the fangs of human teeth, I am ready to allow; but not by inflammatory action in the bone or fang (as swelling of the fang would imply) but by the secretion of ossific matter deposited on the fang, subsequent to the complete formation of the tooth, thereby enlarging it; and this I shall account for in the following manner.

When a tooth is so far decayed, that the attachment of the nerve and blood vessels are broken up and no longer adhere to the canal of the tooth, they contract or retire towards the ends of the roots. In many instances a vesicle is soon after formed at the end of the fang; inflammation succeeds, attended with the most severe pain and throbbing, until the vesicle acquires frequently the size of a large pea.

This, like many other preternatural enlargments, or excrescences, is found on examination in an inflamed state, to be full of blood vessels; and thus it must be supposed that it finds some mode of accommodating itself to the bottom and sides of the socket; as it is impossible to be contained in the socket while it is entire, and in an unimpaired state, for the fangs are closely embraced by it on every side. When the inflammation in this enlarged membrane subsides, which is frequently the case, without coming to a suppuration, the circulation probably becomes languid, and the vessels deposit ossific matter upon the fang of the tooth, until it acquires nearly the size that the excrescence-like substance had previously occupied.—If inflammation should supervene, from whatever cause, at any subsequent period, and on the same root or fang, this excrescence or vesicle again enlarges, and soon finds or makes a passage through the external, and sometimes internal plates of the alveoli.* Swelling and inflammation soon spread through the surrounding integuments, and suppuration follows. When the tooth or fang is extracted in this state, the greater part of the enlarged membrane at the bottom of the fang is destroyed most probably by the presence of acrid pus lodged in the bottom of the socket.

That this enlargement of the fangs of teeth (by bony matter being deposited on them) takes place subsequent to the complete formation of the natural tooth, there remains no doubt. That it is produced in the above manner is equally as conclusive, since in no instance I believe is it to be seen in the human teeth, except when this affection of the membrane has prevailed. I have several of those specimens in my possession, some of which are so much enlarged at the extremity of the fang , as to have been mistaken by professional men for the crown of one of the bicuspedis (which they resemble) while the other, or upper end, being in a state of decay, was supposed to present an instance where the extremity had been absorbed while in the jaw.

The specimens which I allude to, have more the appearance of the crown of a tooth, because the additional bone is compact, and like other solid bone; consequently whiter than the root of the tooth, the extremities of which, where the blood vessels and nerve entered for the support of the tooth, are at the same time plainly to be seen, giving it the appearance of a tooth worn down at one of its points by mastication, until the bone under the enamel is exposed to view.

Lastly, I beg leave to call your attention to a more striking instance where bone is deposited alone by the blood vessels, for no other possible

* This I consider the effect of acrid secretion from the vesicle or inflamed membrane; and analogous to an aneurismal sac, &c.

medium exists by which it can be deposited and formed. I am sensible that this subject needs no further illustration, since authors are agreed on it; but my object is to point out a more probable means by which bone or crusta petrosa is deposited, than by any membrane whatever. The teeth of men, as well as animals, are in most instances duly organized with nerves and blood vessels which terminate in a pulpy substance contained in a cavity in the body or crown of the tooth, proportionate to its size. In the incisores or cutting teeth of the cow, ox, buffaloe, &c. it is very large, in human teeth much less. Those teeth are in all cases subject to being worn down by mastication. In human teeth where the molares are prematurely decayed, or extracted, so that the remaining ones, if any, have no opposition, the action of the inferior jaw is thrown immediately upon the front teeth, and bicupsides; and in this instance, the under teeth, in the course of a few years, wear the upper ones, or those in the superior jaw, all away; so that in numerous instances, they are as even with the gums as if they had been sawed off.

For the animals abovementioned, in proportion as the grinders are worn down, or the food on which they subsist is hard, gritty, &c., so likewise are the cutting teeth worn away. This wearing away of the substance is so rapid in some cases, as to render the teeth extremely susceptible, so that the animal abstains from food; more particularly when they have fed for some time where there is a plenty of sorrel. I mean they are often rendered so susceptible as to make it necessary to cauterize the teeth, to destroy their sensibility.

But no sooner does this wearing away, progress so far as to approach the sensible part of the bone of the tooth, than we see a beautiful display of the paternal care and tenderness of nature in protecting her works from injury; for the vessels in the teeth, begin immediately to deposit ossific matter in their cavity, in order to form a barrier against the injury, the vessels must sustain, as soon as they are exposed, which would be inevitable, was it not for this provision The first appearance of the formation of bone in the cavity, is by a small conical point, which, as the tooth wears away, gradually increases in the form of a sugar-loaf, until the pulp, or vessels are completely obliterated, and the cavity entirely filled up.

It may be said, that ossific matter is deposited by the membrane also. I am sensible that the vessels that organize a tooth, are surrounded by a membrane of the most delicate texture, and which lines the canal of the tooth; and I shall readily show that the bone (at least in this instance) is not deposited by it, nor by any part of the tooth,

If deposited by this membrane, we have much reason to believe that the entire tooth was formed by it, and, that this filling up of the cavity, is only the completion of its office or operation, which has gradually progressed from the first point of ossification on the pulp.

If this were true, there would doubtless have been the most intimate and perfect union of this supervening substance that fills the cavity, and the bone of the tooth, composing one uninterupted mass, which is not the case. This little body of osseous matter that fills the cavity of the tooth, may be easily pushed away when the tooth is split open, and even pushed out by an instrument inserted into the canal of the tooth, which evidently shews that it was deposited and formed, subsequent to the formation and completion of the tooth, and for no other purpose than to preserve the due organization of the tooth or teeth, which would otherwise have been prematurely destroyed by the exposure of the vessels.

This is only one, of innumerable instances, in which we see this maternal care manifested. We see it on the soles of our feet, and those of all four-footed animals destitute of hoofs; in our hands and on our elbows, by the thickening of the skin; on the hinder legs of those animals which often stand erect; as the squirrel, rabbit, hare, &c. Also on the neck and beneath the yoke of the docile and humble ox; and even on the breast-bone of those fowls that sit upon a roost, where is seen a callous thickening, or indurated skin, to protect the subjacent parts from injury.*

Dr. Blake has (if correct) assigned the crusta petrosa to very important use; for he observes "The crusta petrosa is therefore deposited for the membranes to adhere to, *and also to prevent* the sockets from being injured by the violent motion performed in grinding."

There are many animals, on whose teeth and grinders there is but little or no appearance of the crusta petrosa, and yet we see no injury arising from a deficiency of it.

The grinders of the mammoth (if I mistake not) are destitute of the crusta petrosa, and if it can be considered as necessary to shield the sockets from injury in any one instance, if we may judge from the form and size of the jaw and teeth, and the probable efforts they intended to sustain, we must admit that it is necessary in this case.

The tusks of the hippopotamus are sometimes covered with substances resembling the crusta petrosa (at least that portion contained within the jaw) and a person acquainted with those teeth, will always make choice of these in preference to the others, provided the transverse form suits his purpose; for the crusta petrosa is easily removed, and underneath it the enamel remains untarnished, or in general without stains, and beautifully white. At other times we see them almost entirely destitute of any signs of it.

If our opinions may be regulated by the size and form of those tusks, and the corresponding part, and their probable use, it will readily be admitted, that, if the agency of any substance is or can be necessary to screen the sockets from injury, it will also be in this instance.

In general the size and form of the jaws and grinding teeth, of almost all animals, seem to be regulated by the various efforts and degrees of action, they were destined to sustain. The peculiar conformation and

---

How does this tally with the opinion, that aneurismal tumours beat away the bones ?

adaptation of the parts are so admirably constructed and fitted to each other, as to preclude the necessity of any aid to prevent injuries to the adjacent parts.

Hence we are left to consider the disposition of the crusta petrosa on the teeth of different animals, as a characteristic in nature, by which, as in botany, the particular order and class, in the immense chain of animal creation, is easily designated, or pointed out to man.

