# Correction

**Published:** 1841

**Authors:** 


### CORRECTION.

The proof-sheets of Doctor Hayden's article, and his remarks before the Convention of Dental Surgeons, as published in the August and September Numbers of the Journal, were sent to him for correction, but were not returned until after the sheets had been worked off; we however, with pleasure give place to the corrections which he made in the present number.

*Page* 139, 2nd paragraph, 6th line from top, for, and that he terms the crusta petrosa, read, and that *which* he terms, &c.

*Page* 140, 6th paragraph, 3d line, read *deposition* instead of disposition.

*Page* 147, 2nd paragraph, 7th line, for saw, read *jaw.*

*Page* 148, 2nd paragraph, 2nd line, for *semi osseous strata*, read *stratum.* It is in the singular number, and so ought to be in every line where it occurs, which is no less than five times.

*Page* 152, 3d paragraph, 5th line, for canullæ, read *cancellæ.*

*Page* 157, 5th line from top, for disposition, read *deposition.*

